# Exploring the Anticancer Potential of the Multistrain Probiotic Formulation *OxxySlab* in Bladder Cancer Cell Lines

**DOI:** 10.3390/antiox14111282

**Published:** 2025-10-26

**Authors:** Valeria Ciummo, Alessia Ciafarone, Serena Altamura, Francesca Lombardi, Marcella Reale, Maria Grazia Cifone, Benedetta Cinque, Paola Palumbo

**Affiliations:** 1 Department of Innovative Technologies in Medicine and Dentistry, University “G. d’Annunzio”, 66100 Chieti, Italy; valeria.ciummo@phd.unich.it (V.C.); mreale@unich.it (M.R.); 2 Department of Life, Health & Environmental Sciences, University of L’Aquila, 67100 L’Aquila, Italy; alessia.ciafarone@graduate.univaq.it (A.C.); serena.altamura@graduate.univaq.it (S.A.); francesca.lombardi@univaq.it (F.L.); mariagrazia.cifone@univaq.it (M.G.C.); benedetta.cinque@univaq.it (B.C.)

**Keywords:** bladder cancer, muscle-invasive bladder cancer, multistrain probiotic formulation, senescence, oxidative stress, cancer therapy

## Abstract

Bladder cancer (BC), particularly its muscle-invasive subtype (MIBC), remains a clinical challenge due to high recurrence and limited therapeutic options. Emerging evidence suggests that probiotics may offer selective anticancer effects while preserving healthy tissue. In this study, we evaluated the antitumor potential of *OxxySlab*, a multistrain probiotic formulation, in two BC cell lines (T24 and 5637) and a non-tumorigenic urothelial cell line (SV-HUC1). *OxxySlab* lysate dose-dependently inhibited BC cell proliferation, clonogenicity, and migration, while sparing normal cells. Mechanistically, the treatment suppressed epithelial–mesenchymal transition (EMT), induced senescence, and disrupted redox homeostasis in malignant cells. These effects were associated with the induction of oxidative stress and impaired antioxidant defenses. Co-treatment with vitamin C attenuated ROS accumulation and senescence, implicating oxidative stress as a key mediator. Notably, SV-HUC1 cells retained viability and phenotype, confirming the formulation’s selectivity. Overall, these findings support *OxxySlab* as a promising adjunctive strategy in BC therapy, capable of reducing tumor aggressiveness through redox-mediated senescence and EMT inhibition without harming normal urothelial cells.

## 1. Introduction

Bladder cancer (BC) is among the most prevalent malignancies of the urinary tract, with muscle-invasive bladder cancer (MIBC) representing its most aggressive and life-threatening form. Despite advances in surgical and chemotherapeutic approaches, MIBC continues to pose significant clinical challenges due to high recurrence rates, limited treatment options, and poor long-term survival [1]. Its pathogenesis has been associated with environmental exposures, chronic inflammation, and genetic mutations. Recently, oxidative stress and chronic inflammation have emerged as central contributors to bladder carcinogenesis, influencing tumor initiation, progression, and resistance to therapy [2,3]. These processes result from an imbalance between reactive oxygen species (ROS) and antioxidant defense systems, leading to DNA damage, lipid peroxidation, and the activation of oncogenic signaling pathways, which promote tumor growth and immune evasion [4]. Consequently, modulating oxidative stress has emerged as a promising therapeutic strategy, especially through the use of redox modulator compounds that sensitize cancer cells to treatment [5]. Simultaneously, recent advances in microbiome research have highlighted an important, previously overlooked element in bladder cancer biology: the urinary microbiome. Once thought to be sterile, the bladder is now recognized as a dynamic microbial environment, with its composition potentially influencing the onset and progression of disease. A recent systematic review summarizes urobiome–inflammation interactions and identifies potential microbial markers of dysbiosis as diagnostic tools, highlighting the probiotic-based approaches in bladder pathophysiology [6]. Emerging evidence suggests that dysbiosis, characterized by reduced microbial diversity and an abundance of pro-inflammatory bacteria, can modulate immune responses within the bladder microenvironment [7,8]. In cancerous bladder tissues, there is a higher abundance of certain harmful microbes, such as those from the genera Fusobacterium, Acinetobacter, and Escherichia-Shigella, while beneficial microbes like *Lactobacillus* are often depleted [8,9]. These shifts in microbial composition may disrupt epithelial integrity, activate inflammatory pathways, and facilitate tumor development [10]. Importantly, the composition of urinary microbiomes appears to influence therapeutic outcomes. Indeed, microbial profiles can affect patient responsiveness to Bacillus Calmette–Guérin (BCG) immunotherapy and chemotherapeutic agents such as gemcitabine [11]. Additionally, certain bacteria exhibit enzymatic activities able to stimulate the metabolism and detoxification of drugs, potentially reducing their efficacy [12]. This association between microbial metabolism and drug sensitivity highlights the importance of considering microbiome profiles in personalized treatment strategies. Microbial signatures have also shown promise as non-invasive biomarkers, with concepts such as “urinetypes”, microbiota-based classifications similar to gut enterotypes, proposed to stratify patients by risk and guide clinical decisions [7].

The growing recognition of the urinary microbiome’s role in BC pathogenesis has led to increased interest in microbiota-modulating strategies, particularly the use of probiotics. Probiotics, defined as “live microorganisms that, when administered in adequate amounts, confer a health benefit on the host,” have shown immunomodulatory, anti-inflammatory, and antitumor properties in various cancer models [5]. A rising number of preclinical studies and clinical trials are investigating the potential of probiotics as adjunct therapies for bladder cancer, aiming to restore microbiome balance, enhance immune surveillance, and modulate oxidative stress. Lactic acid-producing bacteria such as *Lactobacillus*, *Bifidobacterium*, and *Lactococcus* have been shown to suppress cancer cell proliferation and downregulate pro-inflammatory cytokines [13]. Their presence in the urinary tract may therefore help stabilize the local microenvironment and counteract the dysbiosis associated with tumor progression. Miyake et al. showed that oral administration of *Lactobacillus casei Shirota* and *Bifidobacterium breve* enhanced gemcitabine/cisplatin efficacy in a BC mouse model, reducing tumor size and promoting CD8^+^ T cell activation and dendritic cell recruitment [14]. Also, epidemiological studies support a protective role of probiotics against BC. Ohashi et al. found that habitual intake of lactic acid bacteria reduces the risk of BC [15]. Probiotics may also play a role in preventing recurrence following surgical resection or intravesical therapy. Evidence from animal models and human studies suggests that both orally administered and intravesically delivered probiotics can reduce tumor size, modulate immune responses, and extend disease-free intervals [7]. Though clinical studies remain limited, the favorable safety profile of probiotics and their potential to work in synergy with conventional treatments, such as BCG immunotherapy, make them appealing candidates for further investigation. Notably, interactions between microbes, such as the synergy between *Lactococcus* and *Lacticaseibacillus* observed in treatment-naïve bladder cancer patients, may help stabilize the urinary microbiome and reduce tumor-promoting inflammation [13].

Among the most promising developments in this field is the exploration of multistrain probiotics as adjuncts in BC management. Unlike single-strain formulations, multistrain probiotics combine several beneficial microorganisms to exert synergistic effects on host immunity and microbial balance. Probiotic mixtures aim to restore microbial diversity, inhibit pathogenic overgrowth, and reinforce mucosal barrier integrity, essential factors for maintaining a healthy bladder environment [5,13]. Beyond the direct antitumor effects, probiotics appear to modulate the immune system by enhancing cytokine production, activating dendritic cells, and promoting T-cell responses. These mechanisms may complement existing treatments, such as BCG immunotherapy.

In this framework, *OxxySlab* (also known as SLAB51) is a multistrain probiotic formulation developed by Professor Claudio De Simone. Composed of eight bacterial strains, *OxxySlab* demonstrated antioxidant, anti-inflammatory, and immunomodulatory properties across various models. In human intestinal epithelial cell lines, its lysate forms stabilized HIF-1α via the PI3K/AKT signaling pathway and reduced inflammatory markers, such as NF-κB and IL-1β [16]. Additionally, in different in vitro and in vivo models of neurodegenerative diseases, *OxxySlab* activated SIRT1-dependent pathways, reduced oxidative damage, and improved neuronal survival [17,18]. These results suggest broader applications for *OxxySlab* in conditions where oxidative stress and inflammation play a central role in disease progression. Previous studies have primarily evaluated single-strain probiotics without thoroughly investigating the cellular and molecular mechanisms underlying their effects. In the present work, we tested the *OxxySlab* lysate, heightening for the first time its ability to affect the redox balance, the EMT and senescence, crucial features of BC. Given the growing evidence that specific probiotic strains can exert selective cytotoxic effects against cancer cells, primarily through oxidative stress modulation, apoptosis induction, and suppression of epithelial–mesenchymal transition (EMT), we hypothesized that *OxxySlab* may exert similar effects in BC. This study was therefore designed to evaluate the impact of *OxxySlab* on BC cell viability, EMT marker expression, redox balance, and cellular senescence, with particular attention to its selectivity toward malignant and non-tumorigenic urothelial cells. Specifically, we assessed its effects on proliferation, clonogenicity, migration, ROS production, antioxidant defense systems, and senescence induction. Our findings contribute to the expanding field of probiotic-based cancer research, demonstrating that a multistrain formulation can selectively impair tumor cell function by promoting oxidative stress and senescence beyond their established role in microbiome modulation.

## 2. Materials and Methods

### 2.1. Cell Lines

The human normal uroepithelial cell line SV-HUC1, the T24 high-grade transitional BC cell line, and the 5637 grade II superficial BC cell line, both commonly used as a model for MIBC at different malignant grades, were used in this study. All cell lines were acquired from the American Type Culture Collection (ATCC) and cultured according to the manufacturer’s instructions. SV-HUC1, T24, and 5637 cell lines were cultured in complete media (F-12K, McCoy’s 5a, and RPMI-1640, respectively), each supplemented with 10% fetal bovine serum (FBS), 2 mM L-glutamine, 100 U/mL penicillin, and 100 µg/mL streptomycin, and maintained at 37 °C in 5% CO_2_ and 95% humidity. All reagents and consumables, where not specified, were obtained from Euro Clone (Milan, Italy). Cells were regularly checked for mycoplasma contamination using the N-GARDE Mycoplasma PCR Reagent set (BioCat, Heidelberg, Germany).

### 2.2. Treatments

*OxxySlab*™ multistrain probiotic formulation (produced by EOS2021 Srl, Ardea, Rome, Italy) is a probiotic mixture consisting of *S. thermophilus* (CNCM I-5570), *Bifidobacterium animalis subsp. lactis* (CNCM I-5571), *Bifidobacterium animalis subsp. lactis* (CNCM I-5572), *L. acidophilus* (CNCM I-5567), *L. helveticus* (CNCM I-5573), *L. paracasei* (CNCM I-5568), *L. plantarum* (CNCM I-5569), and *L. brevis* (CNCM I-5566). Briefly, *OxxySlab* formulation was suspended at the concentration of 133 × 10^9^ colony-forming units (CFU) in 10 mL of phosphate-buffered saline (PBS, Euro Clone, West York, UK), centrifuged at 8600× *g*, washed twice, and sonicated (30 min, alternating 10 s of sonication and 10 s of pause) using a Vibracell sonicator (Sonic and Materials, Danbury, CT, USA). Probiotic cell disruption was verified by measuring the absorbance of the sample at 590 nm with a spectrophotometer (Eppendorf, Hamburg, Germany) before and after every sonication step. The samples were then centrifuged at 17,949× *g*, and the supernatants were filtered using a 0.22 µm pore filter (Corning Incorporated, Corning, NY, USA) to remove any remaining whole bacteria. Total protein content was determined by a Bradford assay, using bovine serum albumin (BSA, Sigma-Aldrich, St. Louis, MO, USA) as the standard. Vitamin C (L-ascorbic acid) powder (Sigma-Aldrich) was resuspended in water and used at a concentration of 0.25 mM.

### 2.3. Analysis of Cell Viability and Proliferation

To evaluate the effect of the probiotic lysate on cell viability and proliferation, the cells were exposed to increasing protein concentrations of *OxxySlab* (50, 75, 100, 150 and 200 µg/mL) for 72 h. Cell count was assessed in a Bürker chamber by optical microscopy (Eclipse 50i, Nikon, Tokyo, Japan) using Trypan blue solution (0.04%, final concentration, EuroClone). Untreated cells were referred to as “CNTR” in all experiments. Analysis of viable cells treated with *OxxySlab* was performed by the IncuCyte^®^ Live Cell Imager system (Essen BioSciences, Inc., Ann Arbor, MI, USA). Cell lines were plated in a 96-multiwell culture plate at 1000 cells/well for T24, 2000 cells/well for 5637 and 7000 cells/well for SV-HUC1. Once attached, media were replaced with fresh one containing *OxxySlab* (50–200 µg/mL). Culture plates were positioned into IncuCyte^®^ instrument, and images were acquired every 6 h from 0 to 72 h after treatment. The Incucyte analysis was extended up to 168 h (7 days) to evaluate the *OxxySlab* long-term effect. To measure the proliferation rate, cell confluence was analyzed by IncuCyte ZOOM™ software (2020b, Essen Bioscience, Newark, UK). Two image sets were acquired from several points of the well, using a 10× objective lens, and all the treatment conditions were run in triplicate.

### 2.4. In Vitro Scratch Assay

To evaluate the effect of different concentrations of *OxxySlab* lysate on cell migration, a scratch assay was performed. Cells were plated in multiwell plate and cultured until reaching confluence. Medium was removed, and the cell monolayers were scratched using a 200 µL pipette tip. Subsequently, the cells were washed with PBS and then treated with *OxxySlab* (50–200 μg/mL). The images of cell migration were taken by the inverted light microscope (Eclipse TS 100, Nikon) at 10× magnification at different time points after the injury. The experiments were conducted in triplicate, and six fields were analyzed for each well. The images were analyzed quantitatively while using the standalone TScratch software 1.0 that automatically calculates the portion of area that is occupied by the cells by a mathematical model to calculate the percentage of wound closure.

### 2.5. Colony Formation Assay

The colony formation (clonogenic) assay is an *in vitro* method to assess cell proliferation by measuring the ability of single cells to form colonies after exposure to chemical or anticancer agents, thus evaluating cytotoxic and growth-inhibitory effects [19]. In this study, the assay was used to assess the long-term proliferative capacity of cells following probiotic lysate exposure by measuring their ability to form colonies over time [20]. T24 and 5637 were seeded in 6-well plates at a concentration of 1000 cells/well and were incubated at 37 °C for attachment. Then, cell medium was changed, and cells were treated with *OxxySlab* (50–200 μg/mL) for 72 h. Next, the cells were maintained in a fresh medium until colony formation was observed after 10 days for T24 and 15 days for 5637. Colonies were gently washed, fixed with cold methanol for 20 min, and stained with crystal violet 0.1% in PBS at room temperature for 10 min and air-dried. Images of the colonies were acquired via optical microscopy (Eclipse 50i, Nikon, Tokyo, Japan), and the number of colonies was quantified using ColonyCountJ, a semi-automated plugin for ImageJ 1.54d [20]. The experiment set was carried out twice in duplicate, and histogram plots are the average number from three analyses.

### 2.6. Western Blot

Cells were collected in ice-cold RIPA buffer (Merck KGaA, Darmstadt, Germany) containing a 100 mM protease inhibitor cocktail (Sigma-Aldrich). Protein concentration was determined by a BioRad^TM^ BCA Protein Assay Kit (BioRad, Hercules, CA, USA) to ensure equal protein loading. Total cell lysates (25 μg protein/lane) were separated by 10% SDS-PAGE in reducing conditions with β-mercaptoethanol 5%. Proteins were electroblotted onto 0.45 µm nitrocellulose membranes (BioRad). Following incubation with 5% non-fat dry milk in Tris-buffered saline for 1 h at room temperature, the membranes were incubated overnight at 4 °C with primary antibodies: goat anti-human vimentin polyclonal antibody (Chemicon International, Temecula, CA, USA; dilution 1:100), mouse anti-human E-cadherin monoclonal antibody (Cell Signaling Technology; dilution 1:1000), rabbit anti-human β-catenin monoclonal antibody (Cell Signaling Technology; dilution 1:1000), rabbit anti-human p21 polyclonal antibody (Santa Cruz Biotechnology, Dallas, TX, USA dilution 1:1000); rabbit anti-human phospho-Nrf2 monoclonal antibody (S40) (1:2000, Abcam, Cambridge, UK); rabbit anti-human p16 polyclonal antibody (Santa Cruz Biotechnology, dilution 1:200); mouse anti-human p53 monoclonal antibody (Santa Cruz Biotechnology, dilution 1:1000); mouse anti-human GAPDH monoclonal antibody (Immunological Sciences, Rome, Italy; dilution 1:1000). As secondary antibodies, peroxidase-conjugated anti-rabbit, and anti-mouse IgG antibodies (1:2000 dilution) were purchased from Sigma-Aldrich, and peroxidase-conjugated anti-goat IgG antibody (1:5000 dilution) was purchased from Santa Cruz Biotechnology. The ECL (Thermo Fisher Scientific, Waltham, MA, USA) was used according to the manufacturer’s instructions to detect chemiluminescent signals. Emission was captured using the chemiluminescence documentation system ALLIANCE (UVITEC, Cambridge, UK).

### 2.7. Cell Cycle Analysis by Flow Cytometer

Cell cycle analysis was performed by staining cellular DNA with propidium iodide (PI). T24, 5637 and SV-HUC1 cells treated with probiotic lysate at 75, 100 and 150 µg/mL for 48 h and untreated cells were enzymatically dissociated with trypsin for approximately 10 min at 37 °C and then centrifuged (800× *g* for 10 min at 4 °C). The resulting pellets were washed in PBS and fixed in cold 70% ethanol at 4 °C for 30 min. Fixed cells were transferred to BD plastic tubes (Becton Dickinson, San José, CA, USA), washed twice with cold PBS, and stained with a mixed solution of PI (50 μg/mL), Nonidet-P40 (0.1% *v*/*v*), and RNase A (6 μg/10^6^ cells) (Sigma-Aldrich) for 1 h in the dark at 4 °C. The distribution of cell cycle phases was analyzed using the FACSCalibur flow cytometer (BD Instruments Inc., San José, CA, USA), equipped with the Modfit LT for Mac V3.0 cell cycle analysis software (Beckton Dickinson). Data from 10,000 events per sample were collected and analyzed.

### 2.8. Immunofluorescence Staining

T24 (1000 cells/cm^2^), 5637 (2000 cells/cm^2^), and SV-HUC1 (6000 cells/cm^2^) were grown on coverslips in a 12-well plate and treated as previously reported for 48 h. Successively, the coverslips were fixed with 4% formaldehyde (Carlo Erba Reagents S.r.l., Cornaredo, Italy) for 20 min, permeabilized with 0.1% Triton X-100 (Sigma-Aldrich) for 5 min, and blocked with 3% BSA (Sigma-Aldrich) for 20 min at room temperature. Coverslips were incubated overnight at 4 °C with Ki-67 monoclonal human antibody FITC (1:50, Invitrogen, Waltham, MA, USA), rabbit monoclonal anti-phospho-Nrf2 (S40) (1:200, Abcam, Cambridge, UK) or mouse monoclonal anti-SOD2 antibody (1:200, Sigma-Aldrich) and then stained for 45 min at room temperature with FITC-conjugated goat anti-rabbit IgG (1:1000, Millipore, Darmstadt, Germany) or FITC-conjugated goat anti-mouse IgG (1:1000, Bethyl, Montgomery, AL, USA). The coverslips were mounted with VECTASHIELD^®^ Antifade Mounting Medium with DAPI (Enzo Life Sciences, Lausen, Switzerland) and then examined at 100× magnification with a fluorescent microscope (Eclipse 50i). Nrf2 and SOD2 fluorescent signals (green) were quantified using the ImageJ image analysis tool for 3 fields per condition. The “corrected total cell fluorescence” (CTCF) was evaluated using the formula: integrated density − (cell area × mean background fluorescence). To correct for autofluorescence, the mean background intensity was calculated from three separate background regions in each image.

### 2.9. Senescence Detection

Cellular senescence was assessed in all cell lines by staining for β-galactosidase (β-gal) activity using a kit by Cell Signaling Technology (Danvers, MA, USA). Following treatments, the BC cells were washed with cold PBS and then fixed with a fixing buffer for 15 min at room temperature. After washing twice, BC and SV-HUC1 cells were incubated with a β-gal staining solution containing 5-bromo-4-chloro-3-indolyl β-D-galactopyranoside (X-gal) at 37 °C without CO_2_. The staining solution was removed after 48 h for T24 cells, which were then maintained in PBS. For 5637 cells, the staining was removed after 4 days, and the cells were likewise kept in PBS. Images were obtained using a microscope. Digital images of β-galactosidase–stained cells were randomly acquired at 10× magnification in bright-field mode using a Nikon Eclipse 50i microscope (Nikon Corporation, Tokyo, Japan). For quantification of blue staining intensity, images were converted to 8-bit grayscale and analyzed using ImageJ software (NIH, Bethesda, MD, USA), selecting the entire image area for measurement. The Mean Gray Value was measured to quantify the average staining intensity. Since in 8-bit images lower gray values correspond to stronger blue staining (0 = black, 255 = white), the measured values were inverted by subtracting them from 255 to directly reflect the level of cellular senescence (Inverted Mean Gray Value = 255 − Mean Gray Value) [21]. The resulting values were expressed as “Inverted Mean Gray Value”, proportional to β-gal activity and senescent cell burden.

### 2.10. Fluorimetric Measurement of Intracellular ROS Levels

Intracellular reactive oxygen species (ROS) levels were evaluated using the Dichloro-dihydro-fluorescein diacetate (DCFH-DA) probe, an indicator of oxidative stress in biological systems (Immunological Sciences, Rome, Italy). Inside the cells, the DCFH-DA probe is deacetylated by esterase to generate nonfluorescent compound DCFH. In the presence of ROS, DCFH is oxidized to the fluorescent molecule 2′,7′-dichlorofluorescein. After treating the cells with probiotic lysate at different concentrations for 24 h, the culture medium was replaced with a DCFH-DA solution (25 µM), and the cells were incubated at 37 °C for 30 min. ROS generation was quantified using a VICTORX4™ fluorometer (PerkinElmer, Waltham, MA, USA), with excitation and emission wavelengths set at 488 nm and 535 nm, respectively. Fluorescence values were normalized to cell number and expressed as relative fluorescence units (RFU) per 10^5^ cells.

### 2.11. Real-Time PCR for Telomere Length Measurement

Telomere length was measured in all cell lines, untreated and treated with probiotic lysates for 48 h, via real-time PCR using a ViiA7 sequence detection system. Total DNA was extracted and purified by using the DNA Mini Kit (Qiagen, Hilden, Germany) according to instructions provided by the manufacturer. An amount of 5 µg of each genomic DNA was used to perform the real-time PCR. Real-time quantitative PCR analysis was carried out by SYBR Green dye detection according to the manufacturer’s instructions. Reverse and forward primers were used at a concentration of 0.5 µM, and their sequences were as follows: TEL forward 5′-CGG TTT GTT TGG GTT TGG GTT TGG GTT TGG GTT TGG GTT-3′ and reverse 5′-GGC TTG CCT TAC CCT TAC CCT TAC CCT TAC CCT TAC CCT-3′. In BC cell lines and SV-HUC1 cells, as an internal control for gene expression normalization, the level of 36B4 was measured by following primers: 36B4 forward 5′-CAG CAA GTG GGA AGG TGT AAT CC-3′ and reverse 5′-CCC ATT CTA TCA TCA ACG GGT ACA A-3′. The fold-change quantification of target genes was calculated with the 2^−DDCt^ method.

### 2.12. Statistics Analysis

Statistical analysis was performed while using GraphPad Prism 8.0.2 (GraphPad Software, San Diego, CA, USA). The data were analyzed using a one-way or two-way ANOVA test followed by Dunnett’s or Tukey’s post hoc test. Student’s *t*-test was used to compare treatment groups with the respective control. Data were from independent experiments repeated two or three times and performed in duplicate or triplicate. The results were shown as the means ± SD (standard deviation) or means ± SEM (standard error mean). *p* values less than 0.05 were considered significant.

## 3. Results

### 3.1. OxxySlab Lysate Selectively Inhibits Malignant but Not Normal Bladder Cell Proliferation

**Figure 1:**
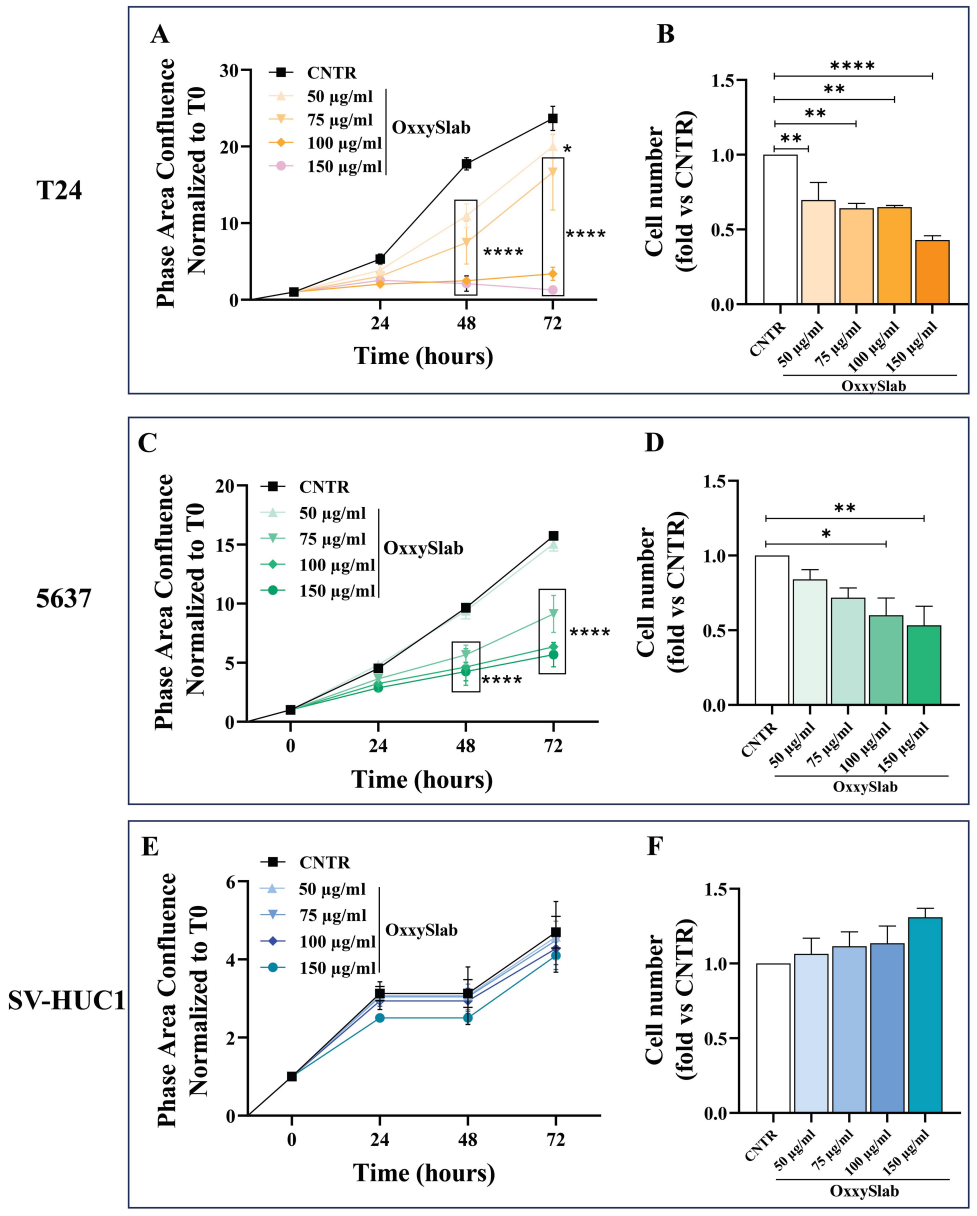

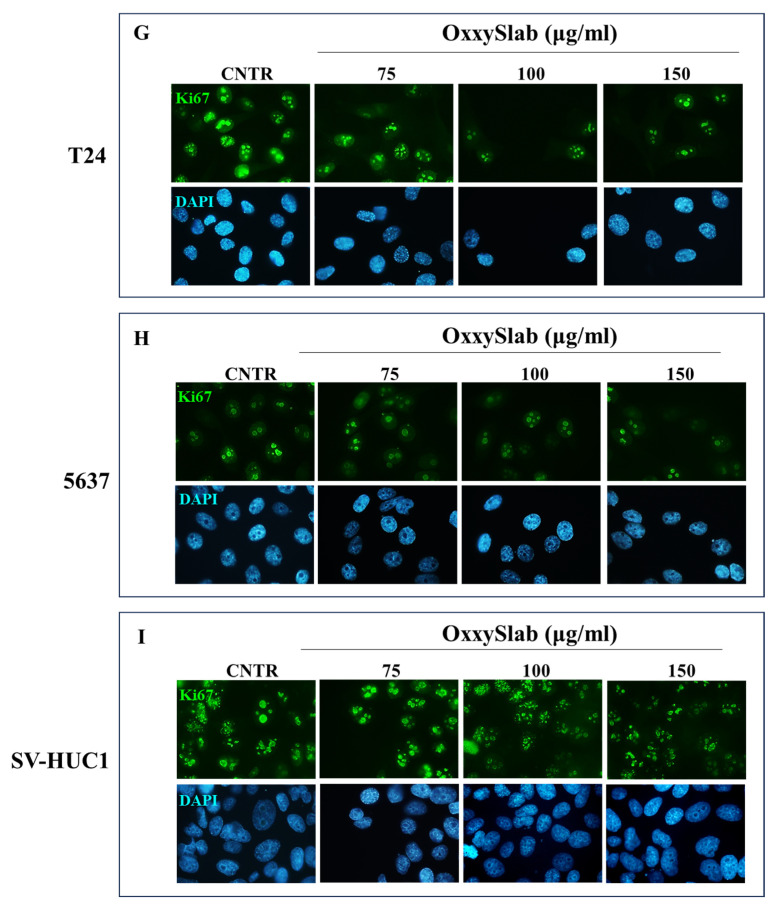
Effect of *Oxxyslab* lysate on proliferation of T24 and 5637, and SV-HUC1 cells. Cells were treated with different concentrations of probiotic lysate (50–200 µg/mL) for 72 h. The proliferation rate, assessed by measuring phase area confluence normalized to time zero (T0), of T24 (**A**), 5637 (**C**) and SV-HUC1 (**E**) was analyzed through the IncuCyte^®^ system and monitored for up to 72 h. The cell number of *OxxySlab*-treated T24 (**B**), 5637 (**D**) and SV-HUC1 (**F**) was assessed by trypan blue dye exclusion test. Data from three independent experiments in triplicate are shown (mean ± SEM). The two-way ANOVA with Dunnett’s post hoc test was used (* *p* < 0.05, ** *p* < 0.01, **** *p* < 0.0001 vs. CNTR). (**G**–**I**) Representative immunofluorescence images of Ki67 (green). Nuclei were counterstained with DAPI (blue). Images were acquired at 100× magnification.

To evaluate the effect of *OxxySlab* lysate on cell proliferation, we exposed BC (T24 and 5637) and non-tumorigenic urothelial (SV-HUC1) cell lines to increasing concentrations of the probiotic lysate. Cell proliferation was assessed by measuring the phase area confluence, normalized to the initial time (T0), using the IncuCyte^®^ Live Cell Imager system. In T24 cells (high-grade MIBC) *OxxySlab* significantly inhibited proliferation in a dose-dependent manner compared to CNTR at concentrations ranging from 75 to 200 µg/mL at both 48 and 72 h (Figure 1A). The cell counts at 72 h confirmed this antiproliferative effect (Figure 1B). A similar trend was observed in grade II MIBC cells (5637). Significant inhibition was evident at concentrations ≥75 µg/mL as early as 48 h, showing a progressive reduction in cell confluence and cell number (Figure 1C,D). In contrast, none of the evaluated concentrations of *OxxySlab* affected the confluence and cell number of SV-HUC1 cells, highlighting the non-toxic effect of the probiotic on normal cells (Figure 1E,F). Hence, *OxxySlab* lysate exerted a selective effect on proliferation, showing strong anti-proliferative activity in both malignant cell lines while leaving normal urothelial cells unaffected. Supporting these results, immunofluorescence analysis of the proliferation marker Ki-67 revealed a gradual reduction in nuclear staining in T24 and 5637 cells exposed to increasing concentrations of the probiotic (Figure 1G,H). Meanwhile, SV-HUC1 cells maintained a strong fluorescence signal even at the highest doses tested (Figure 1I).

To deeply evaluate the long-term effects of *OxxySlab* on the proliferative capacity of BC cells, IncuCyte^®^ analysis was extended up to 7 days post-treatment, confirming a continuous inhibition of proliferation only in T24 and 5637 cells, with no significant changes in SV-HUC1 proliferation (Supplementary Figure S1A). Also, the clonogenic assay demonstrated that probiotic lysate significantly reduced the colony-forming ability of malignant cells in a dose-dependent manner (Supplementary Figure S1B).

### 3.2. OxxySlab Lysate Significantly Delays Wound Closure of Bladder Cancer Cells

**Figure 2:**
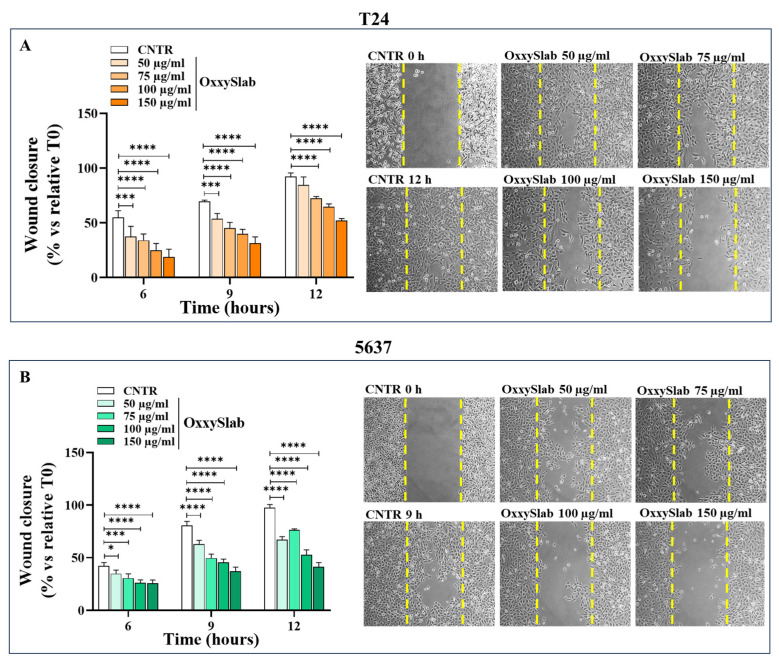

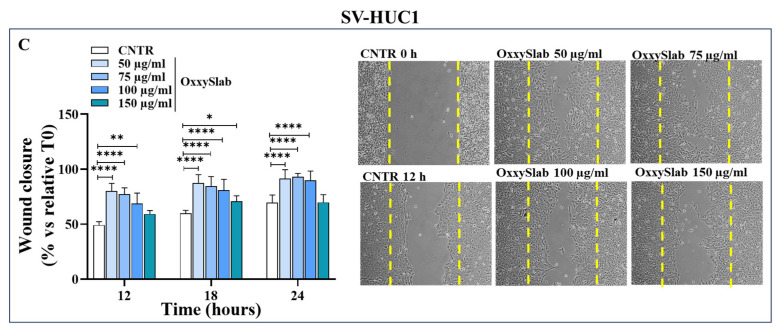
*Oxxyslab* lysate influences proliferation/migration ability of BC cells. Quantitative analysis of wound healing of T24 (**A**), 5637 (**B**), and SV-HUC1 (**C**) cells exposed to increasing *OxxySlab* lysate concentrations. Wound closure was reported as wound closure rate (% to relative T0) of the scratched monolayers. Results are representative of one out of three independent experiments in duplicate and are shown as mean ± SD. Statistical significance was determined by two-way ANOVA followed by Dunnett’s post hoc test (* *p* < 0.05, ** *p* < 0.01, *** *p* < 0.001, **** *p* < 0.0001 vs. CNTR). Representative phase-contrast images (10× magnification) of re-epithelialization of the monolayer are also shown to demonstrate wound closure at the corresponding time points. Initial wound edges at T0 are indicated in each image by yellow dashed lines.

The wound healing is the result of various degrees of cell migration and proliferation depending on the size of the wound, the cell type, the state of the cell cycle and the cellular general metabolic status [22]. The scratch assay is a widely used method for evaluating the proliferation/migratory ability of adherent cells by measuring the closure of an artificial “wound” over time. To assess whether *OxxySlab* lysate could influence the closure ability of our cellular models, the cell monolayers were scratched and subsequently treated with increasing concentrations of the probiotic formulation. Wound closure was monitored at different time points based on the intrinsic characteristics of each cell line. In BC cell lines, which exhibit higher proliferation and migration, typical of high-grade tumor cells, wound closure occurred more rapidly compared to normal cell counterparts. Therefore, the wound closure rate was evaluated at earlier time points (6, 9, and 12 h) for the BC cell lines, while normal cells were assessed at later time points (12, 18, and 24 h) (Figure 2A–C). Untreated (CNTR) T24 cells displayed a nearly complete closure within 12 h. Of note, treatment with *OxxySlab* led to a significant and dose-dependent inhibition of T24 proliferation/migration ability. This effect reached statistical significance as early as 6 h (Figure 2A). A similar trend was observed in the 5637 cells, where each concentration of *OxxySlab* significantly reduced wound closure compared to CNTR as early as 6 h after treatment (Figure 2B). Representative phase-contrast images allowed visualization of the probiotic effect: while untreated cells efficiently filled the gap, cells exposed to the lysate showed a progressively reduced ability to migrate into the scratched area (Figure 2A,B). Higher concentrations of the lysate resulted in a more marked delay in wound closure, suggesting that *OxxySlab* effectively compromised this process in BC cells without negatively affecting it in non-tumoral cells (Figure 2B,C), where it instead accelerated wound closure. This effect was significant at lysate concentrations of 50, 75 and 100 µg/mL (Figure 2C).

### 3.3. Activation of the Epithelial–Mesenchymal Transition in Bladder Cancer Cells Is Counteracted by OxxySlab Lysate Exposure

**Figure 3:**
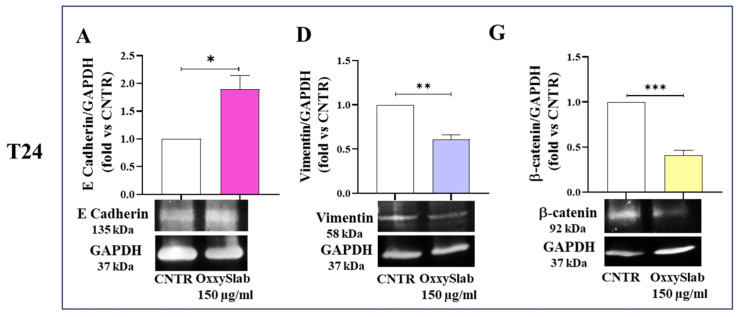

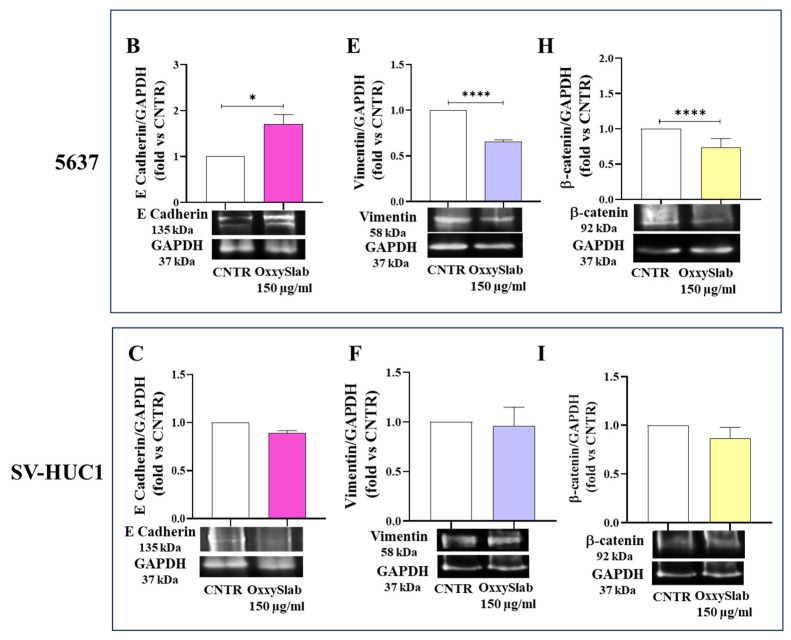
Modulation of EMT markers in BC cells following *OxxySlab* treatment. T24 (**A**,**D**,**G**), 5637 (**B**,**E**,**H**) and SV-HUC-1 (**C**,**F**,**I**) cell lines were exposed to *OxxySlab* lysate (150 µg/mL) for 48 h. Western blot analysis was performed to assess the expression of E-cadherin (**A**–**C**), vimentin (**D**–**F**), and β-catenin (**G**–**I**). Densitometric values were normalized to GAPDH and expressed as fold change vs. untreated control (CNTR). For SV-HUC-1 panels F and I, the same membrane was cut into three parts; therefore, the GAPDH loading control is shared. Data are shown as mean ± SEM of three independent experiments. Statistical significance was assessed using an unpaired Student’s *t*-test (* *p* < 0.05, ** *p* < 0.01, *** *p* < 0.001, **** *p* < 0.0001 vs. CNTR).

Given the critical role of epithelial–mesenchymal transition (EMT) in cancer progression, we investigated whether *OxxySlab* lysate could modulate the expression of EMT-related proteins in our cellular models (Figure 3A,I). In both BC cells, T24 and 5637, treatment with *OxxySlab* at 150 µg/mL resulted in a significant increase in E-cadherin expression compared to CNTR (Figure 3A,B). Moreover, probiotic lysate treatment led to a notable downregulation of vimentin and β-catenin levels in the BC cells (Figure 3D,E,G,H). In contrast, no changes in the SV-HUC1 normal urothelial cells following *OxxySlab* exposure were observed, indicating that EMT-related pathways were not activated in these cells (Figure 3C,F,I). These findings suggest that *OxxySlab* lysate modulates the EMT process specifically in MIBC cell lines by upregulating epithelial markers and downregulating mesenchymal characteristics.

### 3.4. Cell Cycle Modulation Following OxxySlab Exposure

**Figure 4:**
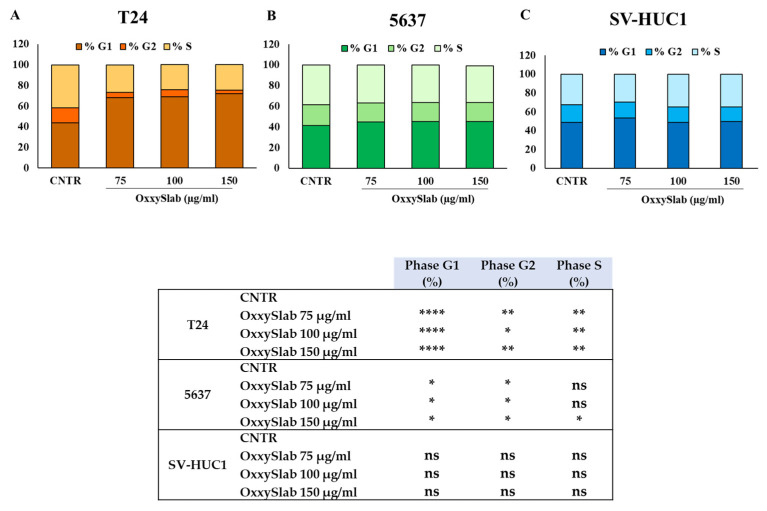
Cell cycle distribution phases following *OxxySlab* treatment. Flow cytometric analysis of cell cycle phases in T24 (**A**), 5637 (**B**), and SV-HUC1 (**C**) cells after 48 h exposure to increasing concentrations of *OxxySlab* lysate. Data are expressed as mean ± SEM of three independent experiments in duplicate. The one-way ANOVA with Dunnett’s post hoc test was used and statistical significance for each cell cycle phase of cell lines is detailed in the corresponding panel (ns = not significant, * *p* < 0.05, ** *p* < 0.01, **** *p* < 0.0001 vs. relative CNTR).

To further investigate the mechanisms behind the antiproliferative effect of *OxxySlab*, the cell cycle distribution was analyzed using flow cytometry. In T24 cells, the probiotic lysate induced a significant accumulation in the G0/G1 phase, indicating effective cell cycle arrest. This effect was consistent even at higher concentrations (Figure 4A). The 5637 cells exhibited a similar response, although to a lesser degree, which remained statistically significant (Figure 4B). In contrast, SV-HUC1 cells showed no significant changes in the distribution of cell cycle phases at any of the tested concentrations, confirming that *OxxySlab* does not affect cell cycle progression in non-tumorigenic urothelial cells (Figure 4C).

### 3.5. Exposure to OxxySlab Lysate Induces Senescence in Bladder Cancer Cells

**Figure 5:**
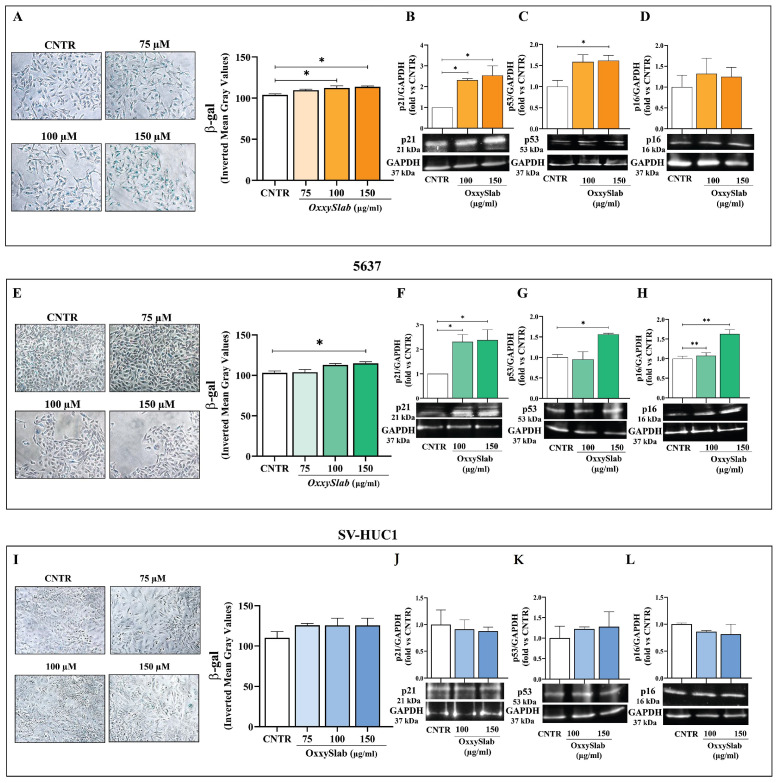
Senescence induction in BC cells following *OxxySlab* lysate treatment. Cell lines were treated with *OxxySlab* lysate (75–150 μg/mL) for 48 h. Senescent cells were identified by β-galactosidase staining (β-gal). Representative phase-contrast images of T24 (**A**), 5637 (**E**) and SV-HUC-1 (**I**) β-gal-positive cells (blue staining) are shown. Quantification of senescence was performed by measuring the Mean Gray Value of blue staining intensity using ImageJ software, with data expressed as Inverted Mean Gray Value (255 − MGV), which directly reflects β-gal activity. Data are expressed as mean ± SEM of two independent experiments in triplicate. Western blot analysis of the senescence markers p21, p53 and p16 was performed in untreated (CNTR) and *OxxySlab*-treated T24 (**B**–**D**), 5637 (**F**–**H**), and SV-HUC1 (**J**–**L**) cells. Protein levels were quantified by densitometry, normalized to GAPDH, and expressed as fold change relative to CNTR of three independent experiments (mean ± SEM). Statistical significance was assessed by one-way ANOVA followed by Tukey post hoc test (* *p* < 0.05, ** *p* < 0.01).

In several cancers, including BC, cellular senescence acts as a physiological barrier against carcinogenesis and tumor progression [23]. To investigate whether the antiproliferative effect of *OxxySlab* was linked to the induction of cellular senescence, we analyzed β-galactosidase (β-gal) activity and the expression of senescence-related markers (p21, p53, and p16) in T24, 5637, and SV-HUC1 cells following treatment (Figure 5). In T24 cells, a significant increase in β-gal staining was observed at 100 and 150 µg/mL (Figure 5A). This effect was accompanied by a marked upregulation of p21 and p53 protein levels (Figure 5B,C), while p16 expression remained unchanged (Figure 5D). Similarly, in 5637 cells, *OxxySlab* induced a dose-dependent increase in β-gal activity (Figure 5E), along with significant upregulation of p21, p53, and p16 (Figure 5F–H). However, *OxxySlab* treatment did not induce senescence in SV-HUC1 cells, as evidenced by the lack of significant changes in β-gal staining and the expression of senescence-associated markers when compared to CNTR (Figure 5I–L). Therefore, it can be hypothesized that *OxxySlab* selectively triggered senescence in BC cells by activating key regulatory pathways, while having no effect on normal urothelial cells.

### 3.6. OxxySlab-Induced Senescence Is Mediated by Oxidative Stress

**Figure 6:**
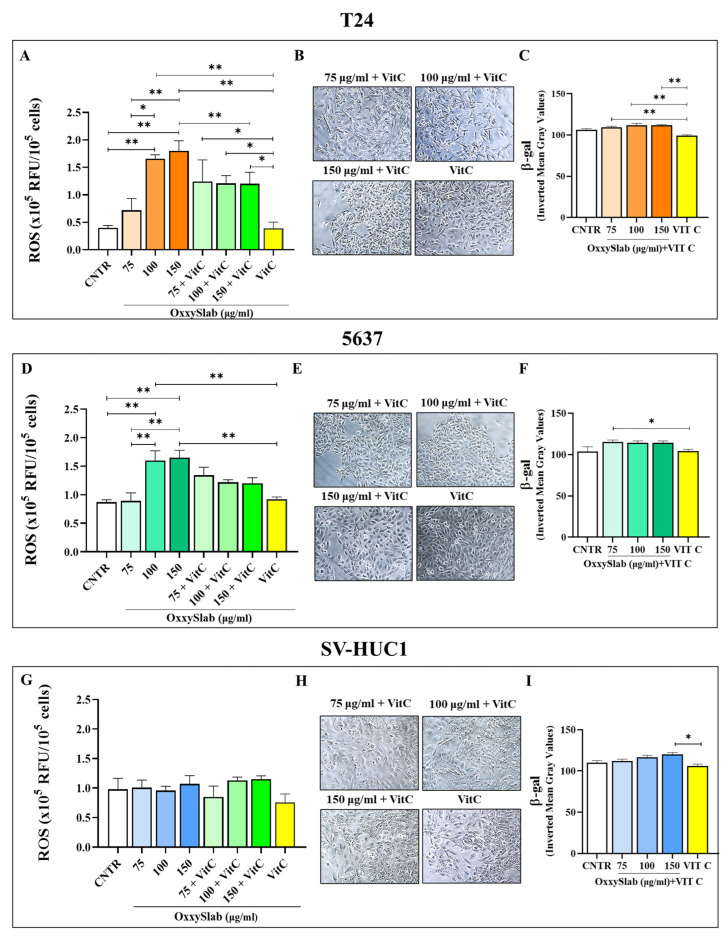
*OxxySlab*-induced senescence is mediated by oxidative stress. T24 (**A**–**C**), 5637 (**D**–**F**) and SV-HUC-1 (**G**–**I**) cells were treated with probiotic lysate alone or in combination with vitamin C. Intracellular ROS levels were assessed by DCFH-DA assay after treatment (**A**,**D**,**G**). Data are expressed as 10^5^ × relative fluorescence units (RFU) per 10^6^ cells and shown as a bar graph of two independent experiments in duplicate (mean ± SEM). Senescent cells were identified by β-gal staining (**B**,**E**,**H**), and quantification was performed by measuring the percentage of β-gal-positive cells (**C**,**F**,**I**). Representative images were taken at 20× magnification. Data are expressed as mean ± SEM of two independent experiments in triplicate. One-way ANOVA with Tukey post hoc test was used (* *p* < 0.05, ** *p* < 0.01).

To clarify the upstream mechanisms underlying the antiproliferative effect of *OxxySlab*, characterized by reduced cell growth, G0/G1 phase arrest, and senescence induction, we investigated whether oxidative stress was involved in triggering these processes. Intracellular ROS levels were measured following treatment, revealing a significant, dose-dependent increase in both T24 and 5637 cells, with maximal ROS production at 150 µg/mL (Figure 6A,D). To confirm whether oxidative stress was responsible for the senescence phenotype, cells were co-treated with the antioxidant vitamin C. ROS levels were markedly reduced in all *OxxySlab*-treated conditions upon vitamin C co-treatment (Figure 6A,D). β-gal staining performed on BC cells co-treated with vitamin C showed a reduction in senescent cell numbers compared to CNTR (Figure 6B,E), which was confirmed by quantitative analysis (Figure 6C,F). These results supported *OxxySlab* as a senescence inducer in BC, with oxidative stress contributing, at least in part, to this effect. In SV-HUC1 cells, *OxxySlab*, even with vitamin C co-treatment, did not induce significant changes in ROS levels or β-gal staining, confirming its tumor-specific activity (Figure 6G–I).

**Figure 7:**
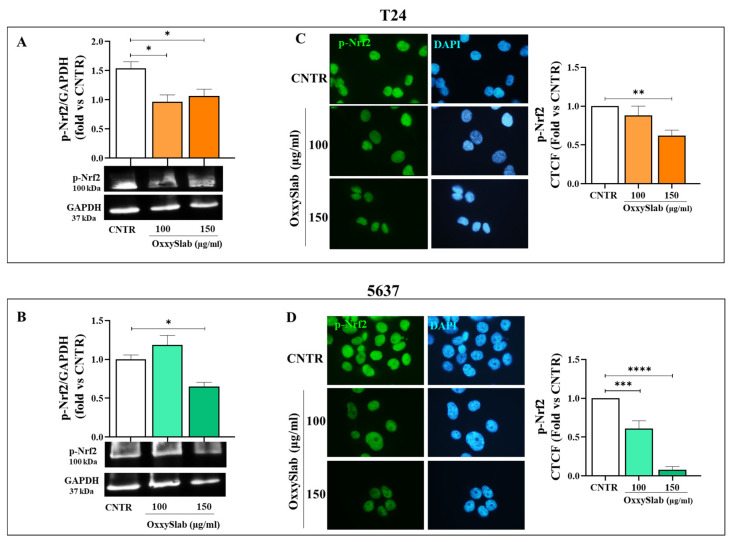

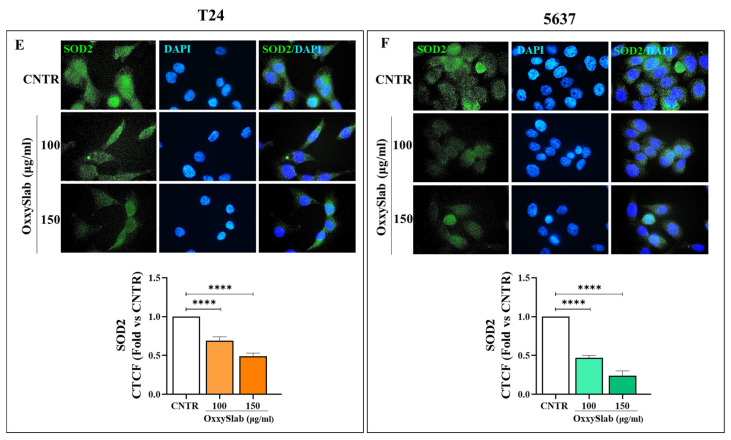
Modulation of antioxidant defense system by *OxxySlab* in BC cells. *OxxySlab*-treated T24 and 5637 cells were analyzed for Nrf2 and SOD2 expression to evaluate antioxidant system modulation through Western blot and immunofluorescence. p-Nrf2 protein was assessed by Western blot in T24 (**A**) and 5637 (**B**); GAPDH was used as loading control. Values represent mean ± SEM of three independent experiments. Immunofluorescence images show the localization of p-Nrf2 (green) (**C**,**D**), and SOD2 (green) (**E**,**F**), respectively. Nuclei were counterstained with DAPI (blue) (100× magnification). Quantification of band intensity and corrected total cell fluorescence (CTCF) values are shown for p-Nrf2 and SOD2. Data are presented as mean ± SEM of three independent experiments in duplicate. One-way ANOVA with Dunnett’s test was applied (* *p* < 0.05, ** *p* < 0.01, *** *p* < 0.001, **** *p* < 0.0001 vs. CNTR).

To thoroughly investigate the role of oxidative stress in the effects induced by *OxxySlab*, we examined the activation status of key antioxidant defense pathways, specifically focusing on p-Nrf2 and its downstream effector, SOD2. These evaluations were conducted in BC cells only, since *OxxySlab* did not induce ROS accumulation or senescence in SV-HUC1 cells (Figure 5I–L and Figure 6G–I), indicating that oxidative stress was not activated in these cells. Our Western blot and immunofluorescence analyses revealed a significant, dose-dependent downregulation of p-Nrf2 in both T24 and 5637 cells following probiotic treatment (Figure 7A,C and Figure 7B,D, respectively). These results indicate that the lysate impairs the antioxidant response in malignant cells, leading to increased ROS accumulation and contributing to the onset of senescence. We then analyzed the expression of SOD2, a key mitochondrial enzyme regulated by p-Nrf2 and involved in ROS detoxification. Immunofluorescence analysis revealed a marked and dose-dependent decrease in SOD2 expression in both T24 and 5637 cells following treatment with *OxxySlab* at 100 and 150 µg/mL (Figure 7E,F). Quantification of fluorescence intensity further confirmed a significant reduction in SOD2 levels compared to CNTR. These findings reinforce the hypothesis that *OxxySlab* undermines the antioxidant defense system in malignant BC, thereby enhancing oxidative stress and promoting senescence.

### 3.7. Selective Telomere Attrition in BC Cells Following OxxySlab Exposure

**Figure 8:**
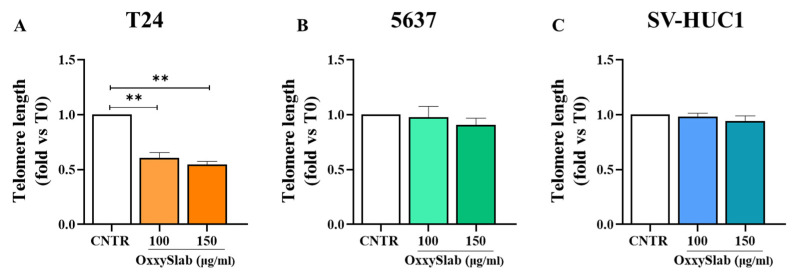
Telomere length analysis following *OxxySlab* exposure. Relative telomere length was evaluated by qPCR in T24 (**A**), 5637 (**B**), and SV-HUC1 (**C**) cells after 48 h treatment with *OxxySlab* at the selected concentrations. Data are expressed as mean ± SEM of three independent experiments. Statistical analysis was performed using two-way ANOVA with Dunnett’s post hoc test (** *p* < 0.01 vs. CNTR).

Given the established link between oxidative stress, cellular senescence, and telomere shortening, we evaluated telomere length following *OxxySlab* treatment. In T24 cells, we observed a significant reduction in telomere length at both 100 and 150 µg/mL. This suggests that *OxxySlab*-induced oxidative stress may accelerate telomere erosion, thereby contributing to senescence (Figure 8A). In contrast, no significant changes in telomere length were detected in 5637 or SV-HUC1 cells (Figure 8B,C). This indicates that the telomere shortening is a cell-type-specific effect, likely dependent on both the intensity of oxidative stress and the aggressiveness of the cellular phenotype. These findings support the idea that *OxxySlab* may contribute to senescence in BC cells through multiple mechanisms, potentially involving redox imbalance and telomere dysfunction.

## 4. Discussion

Despite advancements in treatment strategies, BC, especially MIBC, continues to exhibit high recurrence rates and limited therapeutic options [24]. The search for novel, selective, and well-tolerated strategies has led to increasing interest in microbiota-derived products, including probiotics, which have shown promising anticancer properties in various tumor models. [25,26]. Probiotics may help to reduce cancer incidence and influence its biology through anti-proliferative, pro-apoptotic, immunomodulatory, and anti-metastatic effects. Additionally, they might improve clinical outcomes of patients who assumed these formulations preoperatively as a preventive measure [27,28,29,30].

In this context, our study investigated the effects of *OxxySlab*, a multistrain probiotic formulation, on BC cells and non-tumorigenic urothelial cells. Our findings demonstrate that *OxxySlab* lysate exerts selective cytotoxic effects on T24 and 5637 BC cells, while preserving the viability and phenotype of SV-HUC1 cells. This selectivity is particularly relevant, as it suggests that *OxxySlab* may target malignant cells without compromising healthy tissue. The observed inhibition of proliferation, migration, and clonogenicity in cancer cells supports the hypothesis that *OxxySlab* interferes with key processes involved in tumor progression. In this context, probiotics are gaining attention as complementary tools in cancer therapy due to their selective action against cancer cells while sparing healthy ones. For example, cell-free supernatant from *Lactobacillus rhamnosus GG* induced apoptosis and cell cycle arrest in colorectal (HT-29, Caco-2 cell lines), cervical (HeLa cell line), and liver (HepG2 cell line) cancer cells without affecting normal epithelial cells, an effect mediated by the upregulation of pro-apoptotic (BAX, CASP3, CASP9, TP53) and downregulation of anti-apoptotic (BCL2) genes [31]. Similarly, metabolites produced by *Lactobacillus plantarum* strains reduced the viability of colorectal (HT-29) and breast cancer cells (MCF-7, MDA-MB-231) in a dose-dependent manner, while sparing normal colon fibroblasts (CCD-18Co) and mammary epithelial cells (MCF-10A) [32]. This selective effect could be attributed to the action of bacteriocins and other bioactive compounds interfering with proliferation and survival pathways in cancer cells. Additional evidence supports this selective effect: *Lactiplantibacillus plantarum* Y33 inhibited proliferation and induced apoptosis in oral cancer cells (KB, OSCC) without harming normal cells [33]. This selectivity reflects the high metabolic dysregulation and altered apoptotic mechanisms in the tumor cells, while normal cells preserve the homeostatic control and counteract damage. Also, *Lactiplantibacillus plantarum* reduced the viability of melanoma (A375) and breast cancer (MCF-7) cells by inducing apoptosis, while no significant loss of viability or morphological alterations were observed in normal human keratinocytes. This selective effect was associated with BAX and caspase-3 upregulation, BCL-2 downregulation, and apoptotic morphological changes detected exclusively in tumor cells [34]. However, comprehensive studies evaluating the effects of mixed probiotic formulations on BC cells remain limited.

These *in vitro* findings are complemented by emerging clinical and preclinical evidence supporting the antitumor potential of specific probiotic strains in BC. In BC, certain strains of bacteria, such as those from *Lactobacillus* and *Clostridium* genera, can mimic BCG immunotherapy with similar anti-tumor effects but with fewer side effects. In a clinical study, Naito et al. showed that adding oral *L. casei* to intravesical epirubicin in non-muscle invasive bladder cancer (NMIBC) patients after post-transurethral resection significantly improved 3-year recurrence-free survival (74.6% vs. 59.9% with epirubicin alone), without notable side effects. These results support *L. casei* as a safe and effective adjuvant that may enhance anti-tumor immunity and reduce post-surgical recurrence [35]. Interestingly, Shinnoh et al. demonstrated that *Clostridium butyricum* MIYAIRI 588 exerted antitumor effects in a mouse BC model by stimulating neutrophils to release TRAIL (TNF-related apoptosis-inducing ligand) through the activation of MMP-8 (matrix metalloproteinase-8). TRAIL induced apoptosis in cancer cells without affecting normal ones [36].

Importantly, *OxxySlab* treatment modulated the expression of EMT markers, including a reduction in vimentin and β-catenin and an increase in E-cadherin. These changes suggest a reversal of the mesenchymal phenotype, which is associated with reduced invasiveness and metastatic potential. In human cancers, the Wnt/β-catenin pathway is frequently upregulated, accelerating the malignant behavior of tumor cells. Wnt activation promotes β-catenin accumulation in the cytoplasm, followed by its nuclear translocation, which in turn induces the expression of EMT-related markers and fuels tumor progression and therapy resistance [37]. Thus, the downregulation of β-catenin observed after *OxxySlab* treatment further supports the idea that *OxxySlab* may interfere with transcriptional programs sustaining EMT and tumor aggressiveness. Beyond its impact on EMT, *OxxySlab* lysate induced a robust senescence-like phenotype in BC cells, characterized by increased β-galactosidase activity and upregulation of p21 and p53. Senescence is increasingly recognized as a tumor-suppressive mechanism, capable of halting proliferation in response to cellular stress, DNA damage, or telomere dysfunction. In our model, the induction of senescence was closely linked to oxidative stress, as evidenced by elevated intracellular ROS levels and the attenuation of the phenotype upon co-treatment with vitamin C. This suggests that *OxxySlab* may exploit the redox imbalance typical of cancer cells to trigger a non-apoptotic growth arrest.

The lysate also impaired the antioxidant defense system of tumor cells, reducing the expression of phosphorylated Nrf2 and SOD2, both of which play key roles in maintaining redox homeostasis. This disruption likely contributes to the accumulation of ROS and the downstream activation of senescence pathways. Notably, SV-HUC1 cells maintained their viability and morphology, indicating that *OxxySlab*’s pro-oxidant effects are preferentially directed toward transformed cells. This selectivity may stem from the intrinsic differences in metabolic and redox regulation between normal and malignant urothelial cells.

Interestingly, telomere shortening was observed in T24 cells following treatment, suggesting an additional mechanism by which *OxxySlab* may promote senescence. Telomere erosion is a hallmark of replicative aging and has been implicated in the induction of senescence in various cancer models. The fact that this effect was more pronounced in the highly aggressive T24 line supports the hypothesis that *OxxySlab* may exert stronger pressure on cells with higher proliferative potential and genomic instability. Although senescence is often perceived as a double-edged sword due to its potential to promote chronic inflammation, it is increasingly recognized as a favorable outcome in the context of BC [38,39]. Senescence acts as a tumor-suppressive mechanism by inducing a stable cell cycle arrest in damaged or stressed cells, thereby limiting their progression. This senescence-inducing effect may represent an additional mechanism by which probiotic components inhibit BC cell proliferation.

Taken together, these findings suggest that *OxxySlab* lysate acts through multiple converging mechanisms—including redox imbalance, EMT suppression, and telomere attrition—to impair BC cell viability and invasiveness. The preservation of normal cell function further highlights its therapeutic potential and safety profile.

However, several limitations must be acknowledged. The study was conducted entirely *in vitro*, without considering the complexity of the tumor microenvironment *in vivo*, which may influence *OxxySlab*’s efficacy and selectivity. Moreover, the detailed mechanisms of action need to be clearly defined, given that it is well known that probiotic-derived components, including short-chain fatty acids, peptidoglycans, and bioactive peptides, can influence tumor biology by modulating signaling pathways, oxidative stress, and EMT. To further confirm the link among *OxxySlab*-induced ROS increase, downregulation of the Nrf2/SOD2 axis, and changes in EMT and senescence, future experiments with silencing of Nrf2 and p53/p21 will be performed on MIBC and NMIBC cell models, and we will also use additional ROS scavengers (for example, N-acetylcysteine) and analyze Nrf2 target genes (Heme oxygenase—HO-1). Moreover, the specific bioactive components responsible for the observed effects need to be identified. Future studies should focus on metabolomic profiling of the lysate, validation in 3D cultures and animal models, and exploration of potential synergies with conventional therapies such as cisplatin or immune checkpoint inhibitors.

## 5. Conclusions

This study demonstrates that *OxxySlab* lysate exerts selective antitumor effects on BC cells by impairing proliferation, migration, and clonogenicity, while preserving the viability and phenotype of non-tumorigenic urothelial cells. The formulation modulates key molecular pathways involved in EMT, induces cellular senescence, and disrupts redox homeostasis suggesting a multifaceted mechanism of action. The observed telomere shortening in aggressive tumor cells further supports its potential to limit replicative capacity and tumor progression. Importantly, the selective vulnerability of cancer cells to *OxxySlab*’s pro-oxidant effects highlights its safety profile and translational relevance. These findings position *OxxySlab* as a promising adjunctive strategy in BC therapy, capable of targeting malignant phenotypes through redox-mediated mechanisms without harming healthy tissue.

Future studies should focus on identifying the bioactive components responsible for these effects, validating efficacy *in vivo*, and exploring potential synergies with conventional chemotherapeutics. Overall, *OxxySlab* represents a novel and biologically grounded approach to modulating tumor behavior, with potential applications beyond BC.

We are aware that these results derive from *in vitro* experiments and should be considered preliminary until the *in vivo* validation. From a translational perspective, intravesical delivery of the lysate, rather than oral administration, could appear a valuable and feasible way to achieve high local exposure and then greater efficacy.

## Data Availability

The original contributions presented in this study are included in the article/Supplementary Materials. Further inquiries can be directed to the corresponding author.

## Supplementary Materials

The following supporting information can be downloaded at https://www.mdpi.com/article/10.3390/antiox14111282/s1. Figure S1: Long-term anti-proliferative effect of *OxxySlab* lysate on BC cells. Cells were treated with increasing concentrations of *OxxySlab* lysate (50–200 µg/mL) and monitored up to 168 h (7 days) using the IncuCyte^®^ live-cell imaging system (A). The proliferation rate was assessed by measuring phase area confluence normalized to time zero (T0). Clonogenic assay evaluated in T24 and 5637 after *OxxySlab* treatment was performed, and colony formation was quantified in both malignant cell lines (B). Representative images of crystal violet-stained colonies are shown. Data from three independent experiments are presented as mean ± SEM. Statistical analysis was performed using two-way ANOVA with Dunnett’s post hoc test (* *p* < 0.05, ** *p* < 0.01, *** *p* < 0.001, **** *p* < 0.0001 vs. CNTR).

## Abbreviations

The following abbreviations are used in this manuscript:

BC	Bladder cancer
CFU	Colony Forming Units
CNTR	Control
CTCF	Corrected Total Cell Fluorescence
DCFH-DA	2′,7′-Dichlorodihydrofluorescein Diacetate
EMT	Epithelial–Mesenchymal Transition
β-gal	β-galactosidase
GAPDH	Glyceraldehyde-3-Phosphate Dehydrogenase
MIBC	Muscle-Invasive Bladder Cancer
MGV	Mean Gray Value
Nrf2	Nuclear Factor Erythroid 2–Related Factor 2
RFU	Relative Fluorescence Units
SOD2	Superoxide Dismutase 2
