# Supplementary material for: Exploring the Anticancer Potential of the Multistrain Probiotic Formulation OxxySlab in Bladder Cancer Cell Lines
Source: Antioxidants (Basel). 2025 Oct 26;14(11):1282. doi: 10.3390/antiox14111282 (PMC12649232; doi:10.3390/antiox14111282)

Exploring the Anticancer Potential of the Multi-Strain Probiotic Formulation *OxxySlab* in Bladder Cancer Cell Lines

Valeria Ciummo, Alessia Ciafarone, Serena Altamura, Francesca Lombardi, Marcella Reale, Maria Grazia Cifone, Benedetta Cinque and Paola Palumbo.

Supplementary Figure S1

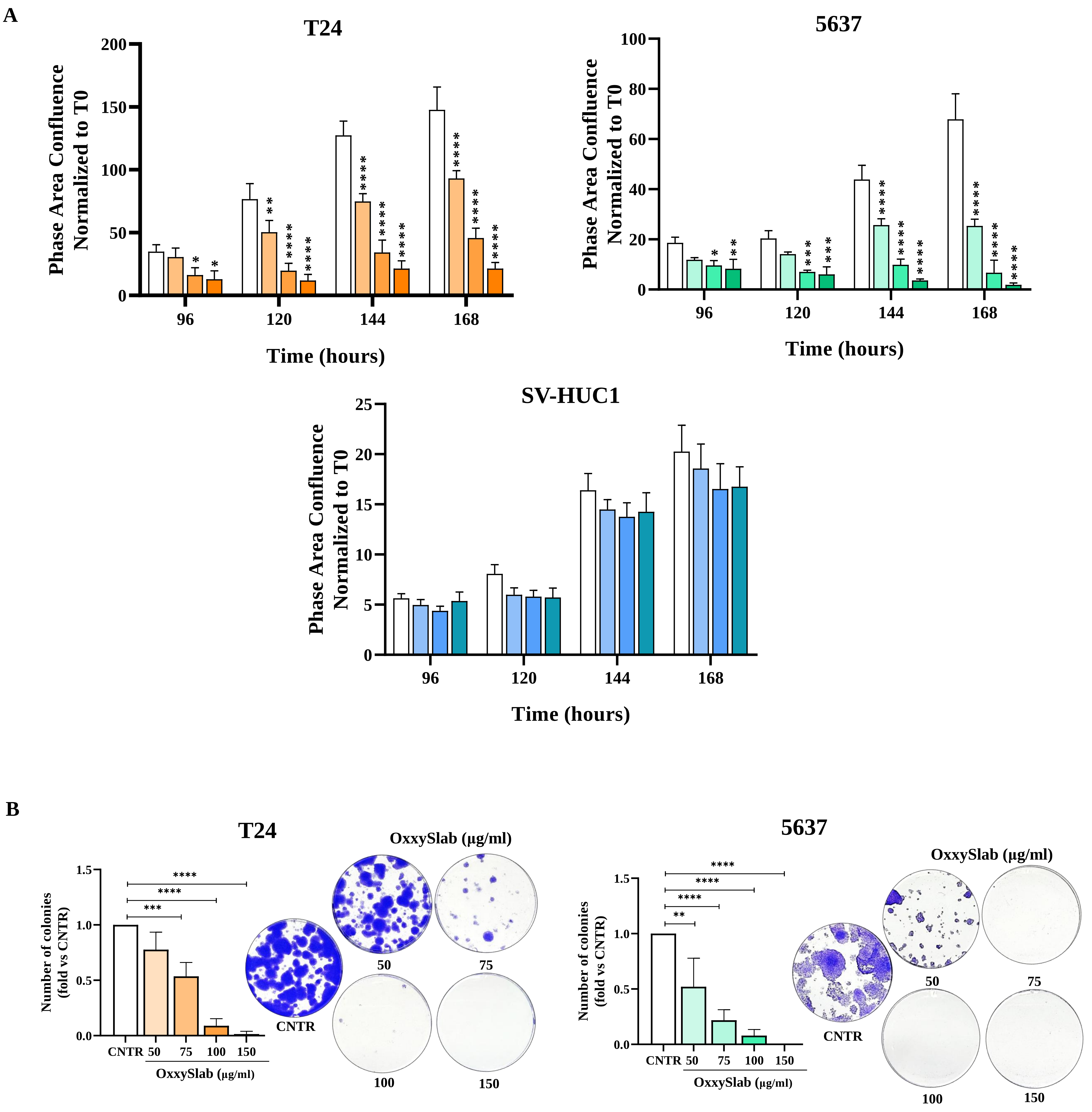

Supplement: Supplementary file 1 [file antioxidants-14-01282-s001.zip › antioxidants-3923716-supplementary.pdf]
